# SOCIAL PARTICIPATION OF OLDER ADULTS AT RISK OF MARGINALIZATION: RESULTS FROM A SCOPING REVIEW OF INTERVENTIONS

**DOI:** 10.1093/geroni/igae098.0100

**Published:** 2024-12-31

**Authors:** Stéphanie Meynet, Trang Nguyen, Alexia Alaux, Melanie Levasseur

**Affiliations:** Université de Sherbrooke, Sherbrooke, Quebec, Canada; Centre de recherche sur le vieillissement, Sherbrooke, Quebec, Canada; Université de Sherbrooke, Sherbrooke, Quebec, Canada; Université de Sherbrooke, Sherbrooke, Quebec, Canada

## Abstract

Considering demographic aging, it is essential to better consider the heterogeneity of older adults in promoting the health of all, especially by fostering social participation of the population at risk of marginalization and social exclusion. Despite vulnerability to isolation and loneliness this population, knowledge about social participation interventions lack integration, and this group is under-represented in studies. This study thus aimed to identify, describe, and integrate knowledge about interventions that foster the social participation of older adults at risk of marginalization and social exclusion. Using 61 keywords in 6 databases, a scoping review identified 24 studies that assessed the influence of interventions on older adults’ social participation, isolation, loneliness, social interactions, and social connectivity. While almost half of the interventions were individualized home-based, the others involved collective activities in the community. Most studies focused on older adults having low income, and only two studies documented interventions with older adults from minority groups of different gender identity or sexual orientation. Interpersonal relationships with peers, and free group activities based on older adults’ interests and life experience were identified as favoring social participation. Perceived isolation, lack of access to transportation and the diversity of older participants represented challenges to successful interventions. While some initiatives were promising, further studies are needed to better develop and evaluate these interventions including assess their efficacy for this population. Providing integrated knowledge on the current initiatives, this exploratory study is the first step towards better crafted interventions that support healthy aging and more inclusive societies.

